# Let’s decide together: Differences between individual and joint delay discounting

**DOI:** 10.1371/journal.pone.0176003

**Published:** 2017-04-20

**Authors:** Diana Schwenke, Maja Dshemuchadse, Cordula Vesper, Martin Georg Bleichner, Stefan Scherbaum

**Affiliations:** 1 Department of Psychology, Technische Universität Dresden, Dresden, Germany; 2 Fakultät Sozialwissenschaften, Hochschule Zittau-Görlitz, Görlitz, Germany; 3 Department of Cognitive Science, Central European University, Budapest, Hungary; 4 Department of Psychology, University of Oldenburg, Oldenburg, Germany; Middlesex University, UNITED KINGDOM

## Abstract

This study addressed the question whether or not social collaboration has an effect on delay discounting, the tendency to prefer sooner but smaller over later but larger delivered rewards. We applied a novel paradigm in which participants executed choices between two gains in an individual and in a dyadic decision-making condition. We observed how participants reached mutual consent via joystick movement coordination and found lower discounting and a higher decisions’ efficiency. In order to establish the underlying mechanism for dyadic variation, we further tested whether these differences emerge from social facilitation or inner group interchange.

## Introduction

Whenever we feel torn between spending money for short-term enjoyment and long-term savings, we frequently devaluate long-term gains in favor of short-term temptations. In everyday life, humans developed a generic strategy to overcome such unwanted temptations: we constitute binding agreements, ranging from friends collectively trying to lose weight to institutionally organized support groups. Although we cultivate this habit intuitively, the question of how two people jointly evaluate delay discounting decisions has not yet been addressed empirically. With this work, we study the potential impact of social collaboration on such tendencies. Therefore we, first, explore discounting decisions in more detail by distinguishing the impulsive dimension from the decisions’ efficiency by comparing individual and dyadic delay discounting on the bases of two separate measurements. Second, we study possible group-specific mechanisms by not only exploring final decisions but by putting a focus on the process of collaborative delay discounting decision-making.

### The impulsive nature of delay discounting

In case of deciding between sooner but smaller or later but larger rewards, we tend to devalue delayed gains and call for swift gratification, a phenomenon referred to as delay discounting. However, the extend of people’s subjective devaluation typically exceeds the prediction made by normative discounting models [1], especially for shorter delays [2]. The inability to resist the tempting one of two alternatives is a central part of non-planning impulsive behavior. Impulsivity, in terms of a multidimensional concept, includes impaired behavioral inhibition and failed judgment of long-term consequences which leads to devaluation of delayed gratification [3]. Delay discounting tasks are therefore considered as valid measures for impulsivity in human behavior [4,5] corroborated by positive correlations between discounting rates and self-reported impulsiveness [3,6,7]. Accordingly, individuals showing impulsive behavior also show higher discounting rates, e.g. children [8], pathological gamblers [9,10] and individuals suffering from substance use disorders [7,11,12]. The connection between impulsivity and discounting also holds on the neural level, as differential neural engagement reflects delay discounting preferences [13,14]. Hence, frontal areas associated with controlled behavior [15], showed higher engagement when subjects chose long-term over short-term options. In contrast, the opportunity of an immediate reward led to a relatively higher engagement in the dopaminergic innervated limbic system, areas associated with impulsive behavior [16,17].

In the broader field of economic preferences, findings support the idea of less impulsive choices due to some sort of social impact. Binary choices between monetary outcomes taken jointly by established couples are less risky than those made by them individually [18]. Groups, confronted with real and hypothetical lottery outcomes, were more likely to choose safe options than individuals [19]. First indications also imply that delay discounting itself can be modified by social context to some extent. That is, choices made on behalf of others are closer to a normative reference and less influenced by impulsivity and emotional response. This pattern occurs in real world choices [20] as well as in classic delay discounting tasks [13,21]. Also, first evidence on the neural level demonstrated neural activity in dopaminergic systems when self-serving choice sets included immediate rewards—except in case of surrogate decision-making [13].

However, there is converging evidence that impulsivity drives short-term oriented choices which social collaboration might manage to successfully overcome. Conversely, the preference of sooner smaller outcomes is not necessarily dysfunctional but even beneficial in some cases [22]. This view is strengthened by evidence showing that how people devaluate future outcomes adapts to ones’ age and income [23–24], the type of decision domain [25] or environmental uncertainty [26]. In view of this consideration, a later larger preference cannot simply be equated with advanced decision-making. While this work claims to study the differences between individual and dyadic delay discounting, we separate the subjective discounting from the efficiency of decision-making by implementing a normative choice reference. With this perspective, we follow a field of research that is relatively separated from delay discounting, but with a long tradition of focusing the social dimension: group decision-making.

### The diversity of processes in group decision-making

Whereas discounting research failed to address the question of group-individual discrepancy, research on a variety of other decision-making tasks demonstrated group advantages compared to the average individual performance of its members, i.e. for problem solving [27,28], reasoning [29], quantity estimation [30,31], perceptual discrimination [32]. Even if in some cases groups suffer losses from unshared information [33], social loafing [34] or coordination difficulties [35], the general effect of group superiority remains undisputed. At least two main lines in research offer reliable explanation for the beneficial effect of social impact on decision-making.

#### Interchange

The conception of additive and interactive processes commonly agree on groups benefitting from the interchange of their members’ diverse perspectives, multiple areas of expertise, a larger pool of information and cognitive resources such as memory or attention [36–38]. With this as a basis, group members achieve the ability to mutually correct their mistakes in the process of finding a decision [27,39], while no such tendency occurs with individuals. Group members also increase their performance on an individual level due to interactive group-to-individual learning processes which leads to higher group performance eventually [29,40].

#### Social facilitation

Alternatively, the theory of social facilitation [41,42] considers the explanatory role of the social context itself. As a consequence of the mere presence of a co-actor, people improve their individual performance of solving simple or well-known tasks. Concluding from sparse evidence obtained from research on real-life choices, social facilitation is not necessarily limited to decision-making, but may also apply to the field of self-control. Studies about food-choices demonstrated that the presence of observing others can decrease the amount of food-intake [43]. The inhibition of an unwanted desire might be facilitated by a socially derived normative expectations [44]. Further, the salience of a social context influences participants regulatory abilities on tasks requiring inhibition and persistence [45].

Taken together, it is well exploited that group decision-making is closer to a normative reference compared to individuals’ decisions. However, it remains unclear whether this phenomenon occurs also in delay discounting and whether it may affect the impulsiveness and the efficiency of delay discounting.

### Our research

In this study we address the question whether social collaboration affects decisions between delayed gains regarding two core dimensions: Based on the findings of risky group choices and surrogate delay discounting, we hypothesize that dyads show less impulsive decision-making and therefore discount less than individuals.

We further argue that based on the general conclusion that group decision-making is closer to a normative reference, dyads would perform more efficiently. Therefore, we used a normative choice model to define each choice as normatively advantageous or disadvantageous [46]. In order to comply with the requirements of judging a decision’s efficiency as a supplemental perspective to traditional discounting parameters, our methodical approach differs in important ways from classic delay discounting. Participants here collected the sooner or later rewards within a real-time reference which led to a trial-by-trial experience of small delays and small rewards in contrast to presenting large delays abstractly in a far future.

Classic delay discounting studies also ask for a choice without an opportunity to look at the process of finding the decision [47,48]. However, this study aimed to gain insight on the possible underlying mechanism, i.e. social facilitation or interchange, which asks for a process-oriented perspective on how two individuals find their common decision. We therefore implemented a new procedure of choice selection allowing us to track the sequences of events at any point during decision-making.

## Methods

### Ethics statement

The study was performed in accordance with the guidelines of the Declaration of Helsinki and of the German Psychological Society. An ethical approval was not required since the study did not involve any risk or discomfort for the participants. All participants were informed about the purpose and the procedure of the study and gave written informed consent prior to the experiment. All data were analyzed anonymously.

### Participants

Sixty students of the Technische Universität Dresden, Dresden, Germany (45 female, mean age = 22.9, SD = 3.6), participated in the experiment. All participants had normal or corrected-to-normal vision. Each group consisted of two participants that were grouped based on their time slot preference yielding 30 two-person groups (18 female-female; 3 male-male; 9 female-male). The participants of 14 groups knew each other before the experiment, the participants the other 16 groups did not know each other.

We calculated the sample size with a priori power analysis under the following assumptions: For statistical tests we assumed repeated measure ANOVAs with *level of decision-making* as within factor, resulting in three repetitions. We expected an effect size of about *η*_*p*_^*2*^ = 0.2 for the effects of interest signaling the differential effect of *level of decision-making*. On a former study [46], which used a comparable paradigm, correlation among repeated measures between *r* = .68 and *r* = .85 were found. Therefore we expected a correlation among repeated measures of *r* = .70 at least. To detect the effect of interest with an alpha error probability of 5%, a power of 80% and an additional 5% of participants to account for experimental loss, our sample size was approximately calculated by N = 30 for two-person groups [49]. Data collection was stopped after this sample was size was reached.

### General procedure

After both participants gave informed consent, they were seated in front of two computer monitors on opposing sides of the laboratory, with the backs towards each other. They were instructed to keep their eyes focused on their own screen and omit any communication with each other, verbally or nonverbally. After participants gave written consent to the experiment and demographic information was received, they were instructed by a standardized tutorial. After the task was completed, each participant was paid according to their actual choices during the experiment as a sum of all decision across both conditions (M = 2.26 euro, SE = 0.02) plus additional 3€ for their time.

### Task procedure

**Fig 1:**
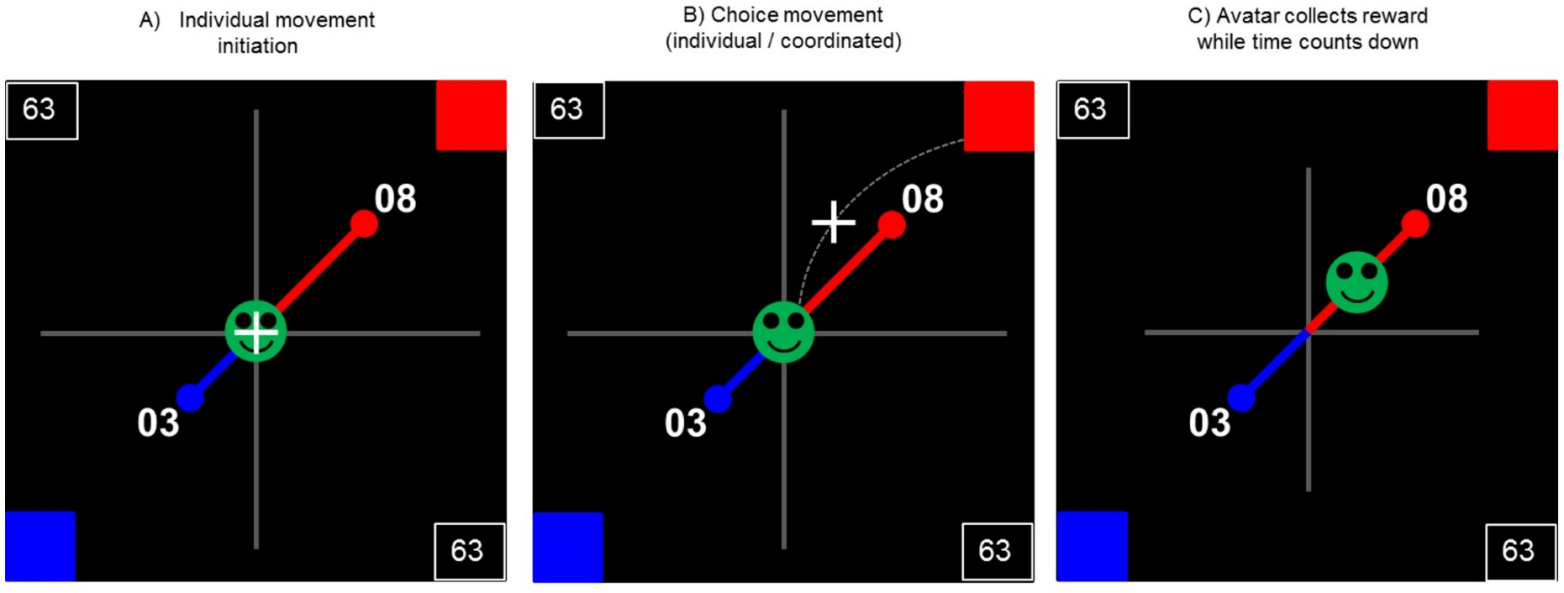
Sketch of the experimental screen and procedure. (A) Each trial started with the presentation of the avatar and the two decision options. The cursor was locked at the center of the screen. The current score participants had already collected was shown in the top/left and the bottom/right corner of the playing area. (B) To select an option, participants navigated the cursor into the color-coded response box in the upper right/lower left corner of the playing area. (C) After reaching a target box, the avatar started to move along a conjunction line from the center of the screen to the place where the chosen option was presented. While the avatar was moving, a limited amount of time was counted down (demonstrated by reducing the length of the grey crossed lines in the background). Because of the farther distance to the avatar, more time was required for collecting the later/larger delivered reward.

Participants’ task was to execute a sequence of choices between a sooner but smaller (SS) or a later but larger (LL) delivered reward. Each trial started with the presentation of the avatar in the center of a computer screen and the two decision-options, one at the upper/right and the other at the lower/left square of the screen (see Fig 1). Each decision value was displayed in numbers, e.g. signaling the monetary value of the option, and connected to the avatar with a diagonal line. Different line lengths indicated varying temporal distance to the avatar, e.g. signaling the temporal delay of the option. Target boxes were presented on the upper/right and the lower/left square of the screen. See S1 File for further information about the apparatus.

To execute their choice, participants had to navigate their cursor via joystick movement into the target box associated with the preferred decision option. We set no time-limit for cursor-navigation to make sure that no time pressure would impact the decision-making process. After reaching a target box, the decision was finally placed. The avatar started to move automatically along the line to the chosen option. While the avatar was moving, the limited amount of collecting-time was counted down, indicated by reducing the size of the two time lines in the background. According to the options’ position and distance to the avatar, more time was needed by the avatar to gain the LL option relative to the SS option. After the avatar had reached the option, the remaining collecting-time refroze and the collected value was credited to the participants account (shown in top/left and the bottom/right corner). The limited time frame prevented the strategy to always choose the SS option in order to finish the experiment earlier. Instead, the experiment lasted a constant amount of time, irrespective of the individual choices.

Between trials, the cursor was locked at the center of the screen during the inter-trial-interval (ITI) of 0.3 seconds. The next trial was started after the ITI when participants had relocated their joysticks to the center position. This prevented that a new decision movement was accidently initiated before the new options were presented.

### Execution of decision-making

**Fig 2:**
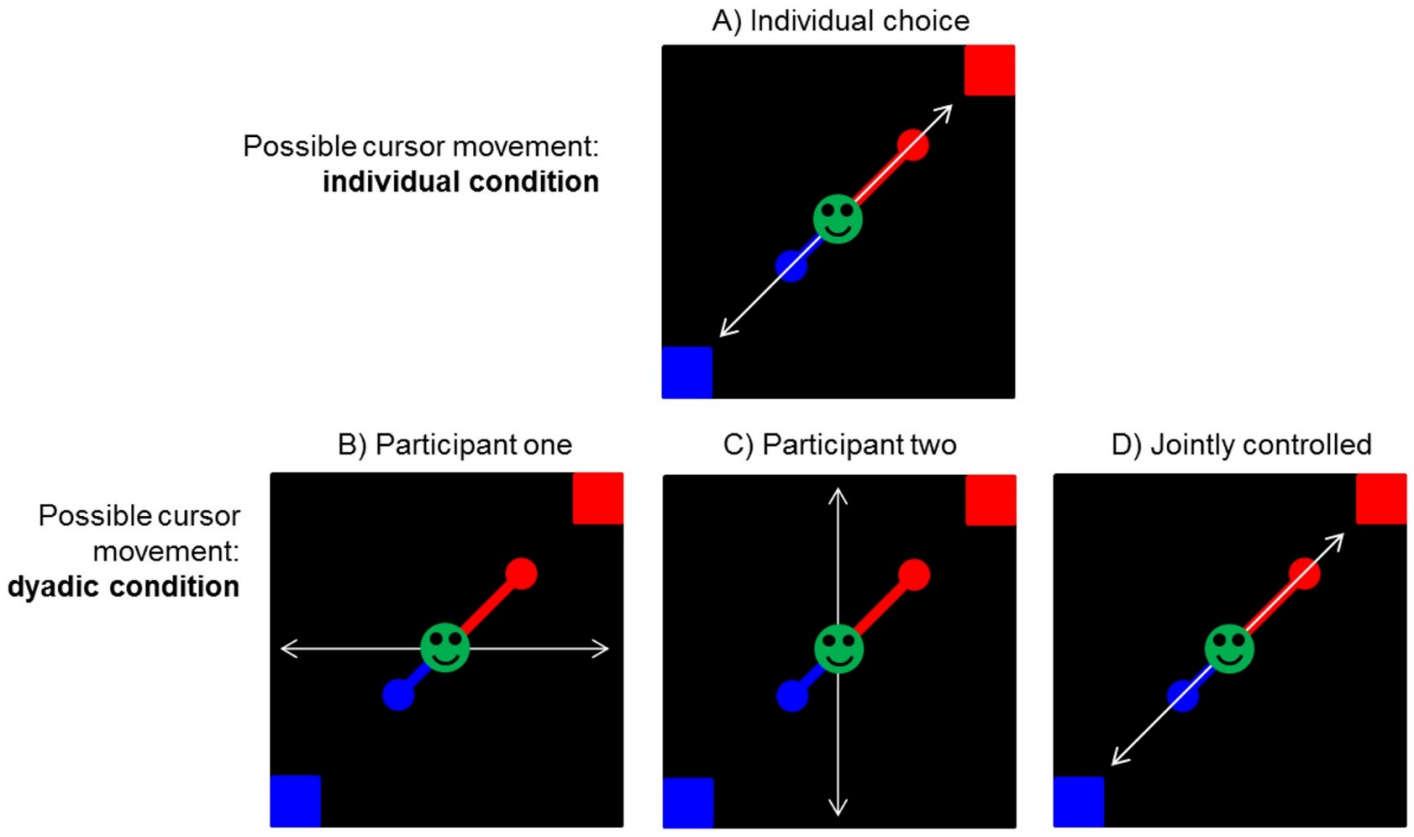
Sketch of the possible cursor movement. (A) In the Individual condition, each participant could move their cursor freely on the screen. Participants therefore could reach their favored target box by executing a diagonal joystick movement. (B & C) In the dyadic condition, movement directions were split up to one participant controlling the vertical movement and the other controlling the horizontal movement while the other dimension was ignored. D) Because both cursor movements were added up, participants were able to move the jointly controlled cursor freely on the screen, comparable to the diagonal joystick movement in the individual condition. The jointly controlled cursor only started moving after both participants crossed an initial threshold of 80% of the maximal possible deflection of their joystick. After initiating cursor movement this way, all amounts of change in joystick movement were effective.

The delay discounting task was executed by each participant in two *conditions of decision-making*: *individual decision-making* in parallel but separated from the other participant of the session and *dyadic decision-making* together with the other participant of the session. When performing their individual choices, participants could reach the favored target box by executing a diagonal joystick movement. In the dyadic decision-making condition, each participant could only move their cursor either horizontally or vertically. To this end both movements were added up together to a diagonal cursor movement (see Fig 2).

To select an option together, participants had to reach final unanimous consent, even if initial cursor movement indicated conflicting preferences of each individual participant. In the case of conflict, the added up cursor movement would move towards the top/left or bottom/right segment of the screen where no target box was located. To end the trial and perform a choice, both participants had to reach mutual consent, only interacting via jointly regulated cursor movement.

By following this procedure, we could distinguish three separate *levels of decision*: (1) the individual decision, which was calculated as the average of both individual decisions within the individual decision-making condition; (2) the pre-decision, which was calculated as the average of both individual decisions within the dyadic decision-making condition by measuring their initial individual joystick-movements; (3) the dyadic decision, which was calculated as participants’ final decision by unanimous assent of both.

Each *condition of decision-making* (individual and dyadic) consisted of four blocks with one block offering 66 seconds collecting-time.

Between blocks (respectively conditions) participants were instructed to rest briefly and to omit any communication. The order of the *condition of decision-making* (individual-dyadic vs. dyadic-individual) was counterbalanced across all two person-groups. The position of the SS option (top/right vs. bottom/left segment) was constant throughout the experiment but counterbalanced across all two person-groups.

### Design

For both *conditions of decision-making*, the options’ values ranged whole numbered from 01 to 05 credits for the SS option and from 06 to 10 credits for the LL option (01 credit = 1/10 € cent), with the SS and the LL value always adding up to 11 credits. The SS option could be reached in 1, 3 and 6 units of time, whereas the LL option could be reached in 2, 3, 4, 5, 7, 8, 9, 12, 14 and 17 units of time (17 units of time = 5 seconds collecting-time). Each small/large pair was combined with each soon/late pair, resulting in a pool of 60 possible types of value-delay combinations (see S2 File for further information about types of value-delay combinations). This pool was replicated 10 times and randomized regarding its order within each replication to ensure that enough trials were available within the given collection-time.

### Data analysis

First, we calculated the extent of discounting by measuring the relative frequency of SS choices and the discounting factor k for each level of decision-making. Second, we calculated the decisions’ efficiency by determining participants’ frequency of advantageous choices. To this end, we classified each trial as an advantageous or disadvantageous choice according to the assumptions of a normative-choice model [46]. With this model, we determined the advantageous choice by comparing value-by-time ratios for both options to identify the option with the higher benefit (see S3 File for further information about the normative choice model).

## Results

We first present the analysis of the extent of discounting followed by the decisions’ efficiency. For the comparison of individual and dyadic decisions, we distinguish three separate *levels of decision*: (1) the individual decision, which was calculated as the average of both individual decisions within the individual decision-making condition. (2) The pre-decision, which was calculated as the average of both individual decisions within the dyadic decision-making condition. (3) The dyadic decision, which was calculated as participants’ unanimous assent.

All measures for the individual decision and pre-decision were aggregated for each individual participant and further averaged over both co-actors in order to avoid inflating statistical power. All statistical results were Greenhouse-Geisser corrected where applicable.

On average, participants completed 181.03 (*SE* = 2.8) trials in the individual condition and 174.25 (*SE* = 3.80) trials in the dyadic condition. Hence, every delay-value combination (60 combinations) was dealt with approximately three times. The first cursor movement after stimulus presentation (RT) took on average 1.60s (*SE* = 0.09s) in the individual and 1.61s (*SE* = 0.06s) in the dyadic decision.

### Measure of discounting

As a measure of discounting, we calculated the relative frequency of choosing the sooner/smaller (SS) instead of the later/larger (LL) option of each participant and each dyad. 42.06% (*SE* = 1.21%) of all trials (i.e. across both conditions) resulted in a SS choice. To check whether individual and dyadic decision-making differed, we performed a repeated-measure analysis of variance (ANOVA) on frequency of SS choices with the factor *level of decision* (individual decision, pre-decision, dyadic decision), yielding a significant main effect (*F*(2, 58) = 6.75, *p* < 0.05, *η*_*p*_^*2*^ = 0.19). Pairwise comparison revealed that participants’ dyadic decision resulted significantly less often in a SS choice (*M* = 40.20%, *SE* = 1.54%) compared both to (1) individual decision (*M* = 43.92%, *SE* = 1.17%), *t*(29) = -2.98, *p* < 0.01, *d* = -0.55, and (2) pre-decision (*M* = 41.63%, *SE* = 1.55%), *t*(29) = -3.15, *p* < 0.01, *d* = -0.58). The difference between individual and pre-decision did not reach significance, *t*(29) = 1.98, *p* = 0.06.

In addition to the frequency of SS choices, we calculated the k value for every level of decision-making and thereby a measure sensitive to the range of delay. Therefore we first identified the subjective indifference for each participant for each delay value. Indifferent points represent the point where both options had the same subjective value e.g. individuals switch from choosing one decision-option to the other one. Therefore we calculated the point of inflection of a logistic function fitted to the participants preferences (SS or LL) as a function of increasing value differences. We then fitted the data to the hyperbolic equation [50,51] and were able to calculate the k value of each individual (individual condition and pre-decision within the dyadic condition) and dyad. With this, we performed a repeated-measure analysis of variance (ANOVA) on the k value with the factor *level of decision* yielding a significant main effect (*F*(2, 58) = 5.66, *p* < 0.05, *η*_*p*_^*2*^ = 0.16). Pairwise comparison revealed a significant smaller dyadic k value (*M* = 0.276, *SE = 0*.*017)* compared to the individual decision (*M* = 0.322, *SE = 0*.*015)*, *t*(29) = -2.80, *p* < 0.01, *d* = -0.512, and the pre-decision (*M* = 0.293, *SE = 0*.*018)*, *t*(29) = -3.82, *p* < 0.001, *d* = -0.70, but no significance difference between individual and pre-decision, *t*(29) = 1.71, *p* = 0.10.

**Fig 3:**
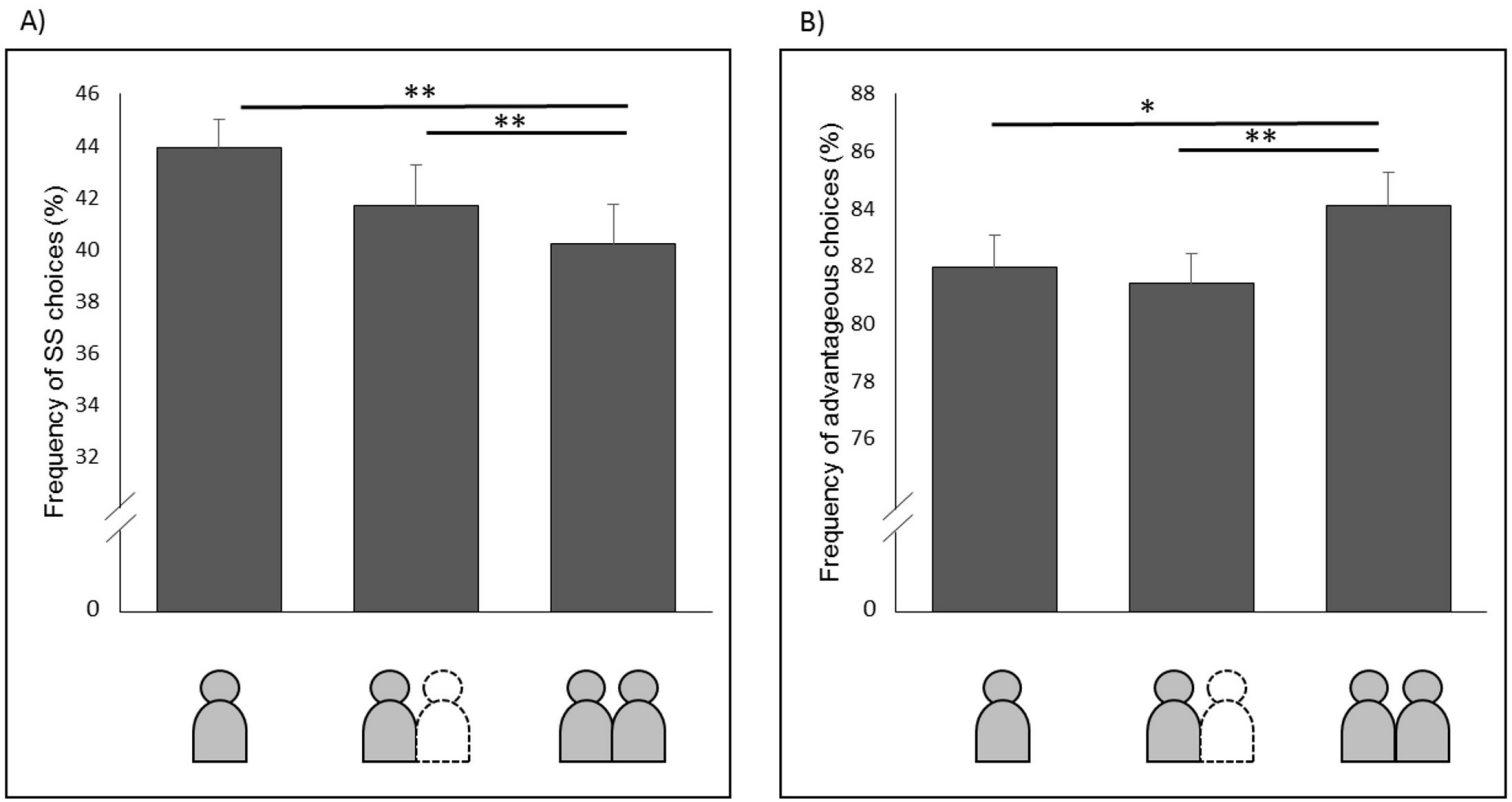
Results. (A) Average frequency of SS choices in % and (B) average frequency of advantageous choices in % depending on the *level of decision-making*, i.e. the individual decision, the pre-decision and the finial common decision are shown. Error bars indicate standard errors of the mean over participants. *Significance at *p* < 0.05 (in comparison to the dyadic decision). ** Significance at *p* < 0.01 (in comparison to the dyadic decision). *** Significance at *p* < 0.001 (in comparison to the dyadic decision).

Hence, as expected, dyadic decision-making led to lower discounting compared to individual decision-making and the initial decision of each participant in the dyadic condition regarding the frequency of SS choices (see Fig 3A) and the discounting parameter k value, a delay sensitive measure.

### Measures of efficiency

To investigate whether participants objectively improved their performance in case of dyadic decision-making, we referred to the classification by the normative-choice model (see Methods). Across all participants and both conditions, the majority of choices could be classified as advantageous (*M* = 83.05%, *SE* = 1.07). We computed an ANOVA on the frequency of advantageous choices with the factor *level of decision* (individual, pre-decision, dyadic decision), yielding a significant main effect, *F*(2, 58) = 10.12, *p* < 0.001, *η*_*p*_^*2*^ = 0.26.

Pairwise comparison revealed that participants made significantly more advantageous choices in the dyadic condition (*M* = 84.14%, *SE* = 1.50%) compared to the individual decision (*M* = 81.96%, *SE* = 1.14%), *t*(29) = 2.74, *p* < 0.05, *d* = 0.50, and the pre-decision (*M* = 81.37%, *SE* = 1.04%), *t*(29) = 6.55, *p* < 0.001, *d* = 1.19, as well. Furthermore, there was no difference between the individual decision and the pre-decision, *t*(29) = 0.85, *p* = 0.40. As expected we found clear evidence, that under terms of deciding together as a dyad, participants’ outperformed individual decision-making concerning both the individual decision-making and the initial decision of each participant in the dyadic condition (see Fig 3B). The comparison of the average gain per trail revealed a slightly higher outcome for dyadic decision-making (*M* = 6.60, *SE* = 0.09) in comparison to the individual condition (*M* = 6.38, *SE* = 0.07), *t*(29) = 3.50, *p* < 0.01, *d* = -0.64.

### Measures of decision process

To provide an understanding of the dyadic process, we next focused on the specific case of conflict-trials. We operationalize conflict as an initial opposed joystick movement, i.e. one player attempted to move to the SS option while the other one attempted to move to the LL option. Overall, 18.20% (*SE* = 0.77%) of all choices were marked as trials with initial opposed preferences.

On average, 42.42% (*SE* = 2.49%) of these trials resulted in a SS choice, indicating that in case of conflicting preferences the dyadic decision finally yielded more LL choices, *t*(29) = —.04, *p* < 0.01, *d* = -0.56 (one-sample t-test against 50%). We measured the time for interaction between both participants (interaction time), as the time starting from first cursor movement to the final decision. In case of a conflict, participants needed on average 3.67s (*SE* = 0.21) to reach a SS while only 3.20s (*SE* = 0.10) to reach a LL decision, *t*(29) = 2.14, *p* < 0.05, *d* = 0.40. Similarly, 84.14% (*SE* = 1.15) of all conflict-trials ended in an advantageous choices instead of a disadvantageous choice, *t*(29) = 28.81, *p* < 0.001, *d* = 5.44 (one-sample t-test against 50%), but interaction time difference did not reach significance, *t*(29) = 1.60, *p* = 0.12.

### Prediction of dyadic decision-making

The difference between individual and dyadic decision-making raises the question if the individual performance can predict dyadic choices. Therefore we calculated Spearman’s correlation, revealing a correlation of *RHO* = 0.72, *p* < 0.001 between individual and dyadic frequency of SS choices respectively *RHO* = 0.74, *p*< 0.001 for frequency of advantageous choices. On the bases of the individual decision-making condition we further compared the frequency of SS choices of each participant with the frequency of his or her co-actor and then grouped each member as either being the relatively *high discounter* or the relatively *low discounter*. In the individual condition the high discounting subjects chose in 48.16% (*SE* = 1.34) of all choices the SS option, while the low discounting subjects only did in 39.67% (*SE* = 1.43). We then calculated Spearman’s correlation between dyadic frequency and individual frequency for each group separately. Both analyses revealed significant correlation, as expectable, but low discounter allowed much better forecasting of the dyadic frequency (*RHO* = .84, *p* <0.001) than high discounter behavior (*RHO* = .48, *p* < 0.01). Similar procedure was accomplished with the frequency of advantageous choices, yielding higher correlation between dyadic frequency and high advantageous group (*RHO* = .79, *p* < 0.001) compared to low advantageous group (*RHO* = .55, *p* < 0.01).

Concluding, dyadic behavior is predictable by individual choices, especially when predication is based on the low discounting and high advantageous participant.

## Discussion

Given the fundamentally social nature of human beings, this study addressed the question whether or not social impact affects delay discounting decisions for the first time. Therefore, we applied a novel paradigm in which participants executed a sequence of choices between two delayed gains in an individual and a dyadic decision-making condition only via joystick movement coordination. We studied whether dyads as opposed to individuals discounted less and performed decision-making more effectively. Further, we aimed a process-oriented perspective on how two individuals find their common decision and tested whether the expected differences emerged from social facilitation or interchange. Our results clearly supported the idea, that dyadic delay discounting differs from individual discounting since dyads showed less discounting and higher decisions’ efficiency. In addition, we identified inner group interchange rather than social facilitation as the causing, underlying process.

### Our findings in relation to existing research

Discounting: Our findings demonstrated less impulsive decision-making for dyadic decision-making since participants chose the LL option in the dyadic condition more often and demonstrated a lower discounting value. This finding is consistent with those from studies showing a beneficial effect of social impact on self-control situations [43] and economic decision-making [19,21]. Still, it must be emphasized that social influence operates in both directions and therefore can lead to higher discounting rates for surrogate delay discounting [52]. Also, there is evidence that neural activation in the medial PFC, which was identified as a value-sensitive region, indicated no differential activity for the frame of references of self-serving versus for other decisions [53,54]. A crucial differential aspect of these studies is the specific procedure under which decision-making took place. Subjects were not asked to decide for but to empathize with someone and therefore to act as if one was somebody else. Consistent with this argument are the observations that trait empathy plays an influential role on surrogate decision-making and that impulsiveness declines with greater distance between decision-maker and beneficiary most probably because of an interpersonal empathy gap [21].

Finally, a promising issue to study in future research is the relationship between impulsivity and prosocial decision-making. There is evidence from situations in which participants apportion a share of hypothetical money between themselves and another participate only acting as recipient. In standard dictator situations, reflected disposers with a lower score in cognitive impulsivity acted more selfish at the expense of the corresponding recipient. Although, reflective in comparison to impulsive dictators were willing to give more money in situations where their own payout was not harmed [55]. A potential similar pattern is indicated by findings of a positive connection between intertemporal patience and generosity towards in-group but not toward out-group receiver [56].

Efficiency: Based on our methodical procedure using a limited time frame to collect the sooner or later options, we provided the key benefit of an objective classification of each trial as being either advantageous or disadvantageous from a normative point of view. With this as a perspective, we were able to transfer the general conclusion of a variety of other group-decision-making tasks to the specific case of delay discounting. Dyadic decision-making was indeed closer to the optimal reference as opposed to individual conducted choices. The character of the paradigm we designed thereby prevents from problems like social loafing [34] and unshared information [33] which could degrade the effectiveness of groups in some cases. Further, quite contrary to the findings of decreased productivity caused by difficulties in coordination [35], the appearance of failed coordination in our study constitutes the principal reason for dyadic success.

### Process of decision-making

Critically, measuring joystick movements allowed us to extract participants’ initial choices within the dyadic setting and compare it to the dyads’ unanimous consent. We found a trend of slightly smaller discounting in the individual pre-decision that did not deviate significantly from individual decision-making. According to findings on social impact on self-control [45] it is reasonable to assume that the exposition to a social context itself facilitated the inhibition of impulsive SS responses. The observing co-actor might activate normative expectations so subjects chose the LL option to live up to this standard [44], but further research is needed to examiner in closer detail. However, since the pre-decision and the dyadic consent significantly differed, we argue that the dyadic lower discounting is mostly attributable to the dyadic interchange. In detail we found conflicting preferences more often to yield in LL than in SS choices. This is in contrast to the simple assumption that two separate preferences of both subjects converge as a compromise, which would lead to conflicting pre-decisions matching the average individual preferences, which is a ratio of 50:50. Since our results contradict such a simple assumption, we hypothesize an interactive error adjusting process [39]: Assuming that co-actor one is attempting to choose the SS option but runs into co-actor two who wants the LL option. Co-actor one might be more willing to re-think his assumingly impulsive first choice and to change his preference relative to his co-actor, while the reverse tendency occurs rarely. This reasoning is also corroborated by differences in interaction time indicating that persuading the SS choosing co-actor to choose the LL option eventually is much easier than the reverse. This point is further strengthened by the finding that dyadic discounting is better predictable by the individual low discounting subject relatively to the high discounting participant indicating an unequal distribution of power or influence of individual preferences. While the same relationship applies to the efficiency of decision-making, with the exception of no variation of interaction time after conflict, further research is needed to shed light on leadership roles in nonverbal decision-making situation.

In conclusion, we assume failed initial cursor coordination—indicating conflict—to induce an adjustment process of communication between both subjects even reduced to a non-verbal joystick movement. Although, interchange in research on group decision-making often relies on verbal communication between group members (e.g. [50]), our results suggest this is not an indispensable requirement. A fine-grained investigation of this action-based negotiation process could be an exciting direction for future research, highlighting e.g. conflict management depending on the composition of the dyad [57], leader and follower roles within dyads [58], the use of cursor movements to exchange communicative signals [59] or insights into the moment-to-moment interpersonal coordination required for efficient joint decision-making.

### A critical evaluation of the paradigm

Due to our aim to measure the interactive process between subjects and to judge decisions efficiency, our experimental paradigm critically differs from classic delay discounting tasks. In contrast to offering much larger decision options, participants here gained the amount of small points of several rounds; in contrast to presenting large time delays abstractly in a far future, we here operationalized small delays within a real-time reference. This procedure leads to a trial-by-trial experience of delay and reward which contrasts future payment after the experiment. The question posed by these methodical alterations is whether our findings are generalizable to discounting behavior observed in more standard delay discounting studies.

Despite the apparent differences, we find our paradigm to be similar in the essence of delays discounting choices, that is, choosing between two options with different outcome and time to achieve. Delay discounting research itself proceeds quite diverse, i.e. using wide ranging time scales from seconds [60] to days [6] to years [61] or directly using delay experience within a trial [60]. This also applies for different kinds of rewards ranging from primary instead of monetary rewards [62] to hypothetical rewards [63,64] to one randomly selected payment after its real-time delay [65,]. Despite the diversity of the research methods, common discounting patterns can be observed in various experimental procedures and types of payment. Empirical evidence of construct validity is also reflected by findings from former work by using a comparable discounting game with also small outcomes and spatial real-time framing [46, 66]. In this work we demonstrated a good correlation of r = 0.64 between k-values measured with this discounting game and a standard intertemporal choice questionnaire [66]. The paradigm used in this study has also been applied to patients suffering from heroin addiction in comparison to controls [67]. Results showed increased discounting for patients compared to controls, matching the results of established measures of discounting, e.g. classic questionnaire approaches [6].

Taking this evidence together, we conclude that our findings are transferable to similar phenomena as in other paradigms aiming to study impulsivity in decision-making. All in all, this work studies the potential differences between individual and jointly conducted discounting choices. In view of the present work, we encourage companionship in order to prevent from impulsive and inefficient decision outcomes.

## Supporting information

S1 File Apparatus. (PDF)

S2 File Types of value-delay combinations. (PDF)

S3 File Normative-choice model. (PDF)

S4 File Data. (CSV)

S5 File Data description. (PDF)
