# Supplementary material for: Let’s decide together: Differences between individual and joint delay discounting
Source: PLoS One. 2017 Apr 20;12(4):e0176003. doi: 10.1371/journal.pone.0176003 (PMC5398579; doi:10.1371/journal.pone.0176003)
Supplement: S1 File — (PDF) [file pone.0176003.s001.pdf]

Let's decide together: differences between individual and joint delay discounting

Supplement Materials S1

Diana Schwenke[1], Maja Dshemuchadse[2], Cordula Vesper[3], Martin Bleichner[4], Stefan Scherbaum[1]

[1]Department of Psychology, Technische Universität Dresden, Dresden, Germany

[2]Fakultät Sozialwissenschaften, Hochschule Zittau-Görlitz, Görlitz, Germany

[3]Department of Cognitive Science, Central European University, Budapest, Hungary

[4]Department of Psychology, University of Oldenburg, Oldenburg, Germany

Correspondence should be addressed to:

E-mail: diana.schwenke@tu-dresden.de (DS)

Apparatus

Stimuli were presented on a black background on two 17-inch CRT screens running at a resolution of 1280 x 1024 pixels (72 Hz refresh frequency). On this screen (see Figure 1), the squared playing area of 1024 x 1024 pixels was marked by a white border. In the center of the screen, an avatar (diameter 26 pixels) was shown, connected with two diagonal lines with different line length (red/blue line of 3 pixels width). At each end of these lines, the value of the two alternative options was presented in white font (Arial, 24pt). Because of the different length, the options' distance to the center / the avatar varied from 20 pixels (indicating 1 unit of temporal distance) to 341 pixels. Two target boxes were presented at the upper/right and lower/left corner of the screen. They were marked by filled red/blue squares of 75 x 75 pixels.

For stimulus presentation we used Psychophysics Toolbox 3 in MATLAB R2010b (MathWorks Inc., Natick, MA) running on two Windows XP SP2 personal computers. Participants executed their choices via two Thrustmaster, T. Flight joysticks while wearing noise-shielding headphones.
