# Supplementary material for: Let’s decide together: Differences between individual and joint delay discounting
Source: PLoS One. 2017 Apr 20;12(4):e0176003. doi: 10.1371/journal.pone.0176003 (PMC5398579; doi:10.1371/journal.pone.0176003)
Supplement: S2 File — (PDF) [file pone.0176003.s002.pdf]

Let's decide together: differences between individual and joint delay discounting

Supplement Materials S2

Diana Schwenke[1], Maja Dshemuchadse[2], Cordula Vesper[3], Martin Bleichner[4], Stefan Scherbaum[1]

[1]Department of Psychology, Technische Universität Dresden, Dresden, Germany

[2]Fakultät Sozialwissenschaften, Hochschule Zittau-Görlitz, Görlitz, Germany

[3]Department of Cognitive Science, Central European University, Budapest, Hungary

[4]Department of Psychology, University of Oldenburg, Oldenburg, Germany

Correspondence should be addressed to:

E-mail: diana.schwenke@tu-dresden.de (DS)

Types of Value-Delay Combinations

For both *conditions of decision-making*, we generated trials according to the following scheme. The options' values ranged whole numbered from 01 to 05 credits for the SS option and from 06 to 10 credits for the LL option (01 credit = 1/10 € cent), with the SS and the LL value always adding up to 11 credits (small/large pairs were: 1/10, 2/9, 3/8, 4/7, 5/6). The SS option could be reached in 1, 3 and 6 units of time, whereas the LL option could be reached in 2, 3, 4, 5, 7, 8, 9, 12, 14 and 17 units of time (17 units of time = 5 seconds collecting-time). Each small/large pair was combined with each soon/late pair, resulting in a pool of 60 possible types of value-delay combinations. This pool was replicated 10 times and randomized regarding its order within each replication to ensure that enough trials were available within the given collection-time.

Table 1: Value-Delay Combinations

Sooner Smaller Option (SS)			Later Larger Option (LL)		
Value*	Time**	Value-by-Time Ratio	Value*	Time**	Value-by-Time Ratio
1	1	1	10	2	5
2	1	2	9	2	4,5
3	1	3	8	2	4
4	1	4	7	2	3,5
5	1	5	6	2	3
1	1	1	10	3	3,33333333
2	1	2	9	3	3
3	1	3	8	3	2,66666667
4	1	4	7	3	2,33333333
5	1	5	6	3	2
1	1	1	10	7	1,42857143
2	1	2	9	7	1,28571429
3	1	3	8	7	1,14285714
4	1	4	7	7	1
5	1	5	6	7	0,85714286
1	1	1	10	12	0,83333333
2	1	2	9	12	0,75
3	1	3	8	12	0,66666667
4	1	4	7	12	0,58333333
5	1	5	6	12	0,5
1	3	0,33333333	10	4	2,5
2	3	0,66666667	9	4	2,25
3	3	1	8	4	2
4	3	1,33333333	7	4	1,75
5	3	1,66666667	6	4	1,5
1	3	0,33333333	10	5	2
2	3	0,66666667	9	5	1,8
3	3	1	8	5	1,6
4	3	1,33333333	7	5	1,4
5	3	1,66666667	6	5	1,2
1	3	0,33333333	10	9	1,11111111

2	3	0,66666667	9	9	1
3	3	1	8	9	0,88888889
4	3	1,33333333	7	9	0,77777778
5	3	1,66666667	6	9	0,66666667
1	3	0,33333333	10	14	0,71428571
2	3	0,66666667	9	14	0,64285714
3	3	1	8	14	0,57142857
4	3	1,33333333	7	14	0,5
5	3	1,66666667	6	14	0,42857143
1	6	0,16666667	10	7	1,42857143
2	6	0,33333333	9	7	1,28571429
3	6	0,5	8	7	1,14285714
4	6	0,66666667	7	7	1
5	6	0,83333333	6	7	0,85714286
1	6	0,16666667	10	8	1,25
2	6	0,33333333	9	8	1,125
3	6	0,5	8	8	1
4	6	0,66666667	7	8	0,875
5	6	0,83333333	6	8	0,75
1	6	0,16666667	10	12	0,83333333
2	6	0,33333333	9	12	0,75
3	6	0,5	8	12	0,66666667
4	6	0,66666667	7	12	0,58333333
5	6	0,83333333	6	12	0,5
1	6	0,16666667	10	17	0,58823529
2	6	0,33333333	9	17	0,52941176
3	6	0,5	8	17	0,47058824
4	6	0,66666667	7	17	0,41176471
5	6	0,83333333	6	17	0,35294118

*1 value units ≙1/10 € cent

**17 time units ≙ 5 seconds collecting time
