# Supplementary material for: Let’s decide together: Differences between individual and joint delay discounting
Source: PLoS One. 2017 Apr 20;12(4):e0176003. doi: 10.1371/journal.pone.0176003 (PMC5398579; doi:10.1371/journal.pone.0176003)
Supplement: S3 File — (PDF) [file pone.0176003.s003.pdf]

Let's decide together: differences between individual and joint delay discounting

Supplement Materials S3

Diana Schwenke[1], Maja Dshemuchadse[2], Cordula Vesper[3], Martin Bleichner[4], Stefan Scherbaum[1]

[1]Department of Psychology, Technische Universität Dresden, Dresden, Germany

[2]Fakultät Sozialwissenschaften, Hochschule Zittau-Görlitz, Görlitz, Germany

[3]Department of Cognitive Science, Central European University, Budapest, Hungary

[4]Department of Psychology, University of Oldenburg, Oldenburg, Germany

Correspondence should be addressed to:

E-mail: diana.schwenke@tu-dresden.de (DS)

Normative-Choice Model

We determined for each trial of each participant in the individual decision-making and dyadic decision-making condition the advantageous choice according to the assumptions of a normative-choice model:

$$\frac{value_{LL}}{time_{LL}} \lessgtr \frac{value_{SS}}{time_{SS}}$$

According to this model, we determined the advantageous choice by comparing the value-by-time ratios for the sooner/smaller (SS) and the later/larger (LL) option to identify which option yielded more benefit. The value was defined by the amount of credits participants could have achieved by choosing an option. As time, we took the units of time in seconds the avatar needed to move to the place where a chosen option was presented. By comparing value-by-time ratios as per description above, each decision option could be classified as either being advantageous or disadvantageous. According to this procedure, in 31 of all generated trials the LL option was classified as the advantageous choice (51.66%). In 29 of all generated trials the SS option was classified as the advantageous choice (48.34).

In case of always choosing the SS option, participants would earn 1617 points in comparison to 1723 points if always choosing LL and 2638 points if always choosing the advantageous option.
