# Supplementary material for: Let’s decide together: Differences between individual and joint delay discounting
Source: PLoS One. 2017 Apr 20;12(4):e0176003. doi: 10.1371/journal.pone.0176003 (PMC5398579; doi:10.1371/journal.pone.0176003)
Supplement: S5 File — (PDF) [file pone.0176003.s005.pdf]

Term	Description	Measure of
RatioSS	Frequency SS advantageous choices	discounting
kValue	K value	discounting
RatioAdv_*	Frequency of advantageous choices	efficiency
RatioSS_conflict_*	Frequency of SS choices in trials with conflict	decision process
IT_CONF_SS	Interaction time in trials with conflict & SS choice	
IT_CONF_LL	Interaction time in trials with conflict & LL choice	
IT_CONF_ADV	Interaction time in trials with conflict & advantageous choice	
IT_CONF_DIS	Interaction time in trials with conflict & disadvantageous choice	

*** for subject assignment to:**

1s = co-actor 1 in the individual condition; 2 = co-actor 2 in the individual condition

1pre = co-actor 1 pre-decision within the dyadic condition, 2pre = co-actor 2 pre-decision within the dyadic condition

pair = final pair decision
